# LncRNA MALAT1’s role in the development of retinopathy: A review

**DOI:** 10.1097/MD.0000000000041954

**Published:** 2025-03-21

**Authors:** Gukun Yang, Xionggao Huang

**Affiliations:** a Department of Ophthalmology, The First Affiliated Hospital of Hainan Medical University, Haikou, Hainan Province, PR China; b Key Laboratory of Emergency and Trauma of Ministry of Education, The First Affiliated Hospital of Hainan Medical University, Haikou, Hainan, PR China.

**Keywords:** EMT, long noncoding RNAs, MALAT1, ophthalmic disease, signaling pathways

## Abstract

Long noncoding RNA (lncRNA) metastasis-associated lung adenocarcinoma transcript 1 (MALAT1) and retinopathy are 2 distinct yet interconnected areas of research in the field of ocular studies. MALAT1, with its diverse biological functions, has been extensively studied and demonstrated to play a role in various diseases, including ocular pathologies. Its involvement in alternative splicing regulation, transcriptional control, and the competing endogenous RNA (ceRNA) network suggests its potential implication in retinopathy. Retinopathy refers to a group of disorders that affect the retina, leading to vision impairment and, in severe cases, even blindness. These conditions include diabetic retinopathy, retinoblastoma, proliferative vitreoretinopathy, retinopathy of prematurity, and retinal neurodegeneration. The understanding of the molecular mechanisms underlying the development and progression of retinopathy, along with the potential involvement of MALAT1, can provide valuable insights for the diagnosis and treatment of this condition. Retinopathy, characterized by various manifestations and underlying mechanisms, presents a significant challenge in the field of ophthalmology. As a complex disease, its pathogenesis involves multifactorial factors, including angiogenic dysregulation, inflammatory responses, oxidative stress, and cellular signaling abnormalities. The emerging role of long noncoding RNA MALAT1 in retinopathy has attracted considerable attention. MALAT1 has been found to participate in multiple cellular processes, including alternative splicing regulation and transcriptional control. Additionally, the competing endogenous RNA (ceRNA) network involving MALAT1 indicates its potential relevance as a regulator in retinopathy. Further investigations into the specific mechanisms underlying MALAT1’s involvement in retinopathy pathogenesis may provide valuable insights into the development of diagnostic and therapeutic approaches for managing retinal disorders.

## 1. Introduction

Noncoding RNAs (ncRNAs) play a crucial role in human health and pathology. The hairless gene has been extensively studied in laboratory rodents and humans, providing insights into skin physiology, aging, drug activity, and toxicity.^[1]^ In the context of cardiovascular health, computational models of the human left ventricle have been developed to understand heart function in diastole, emphasizing the importance of structure-based finite strain modeling.^[2]^ Additionally, LIN28/LIN28B has emerged as an oncogenic driver in cancer stem cells, highlighting the clinical significance of ncRNAs in cancer progression.^[3]^ Furthermore, the colonic axis in chronic kidney disease has been explored, revealing profound changes in the microbiome composition and colonic structure and function due to chronic kidney disease.^[4]^ Islet inflammation in type 2 diabetes has also been investigated, shedding light on the cellular and molecular mechanisms underlying β cell dysfunction and the role of macrophage polarity shift in type 2 diabetes pathology.^[5]^ Moreover, hyperpolarized magnetic resonance imaging has shown promise in assessing molecular processes underlying changes in cardiac function, providing new insights into cardiovascular health.^[6]^ Sex differences in cardiovascular ncRNA research have been a focus of recent studies, with discussions on genetic, epigenetic factors, and sex hormone regulation of transcription in cardiovascular disease.^[7]^ Finally, hydrogel strategies for female reproductive dysfunction have been explored to address infertility issues, highlighting the need for improved treatments and translational research in human reproductive physiology.^[8]^ These studies collectively underscore the importance of understanding the structure, function, and clinical significance of ncRNAs in human health and pathology.

Retinopathy and long noncoding RNA (lncRNA) metastasis-associated lung adenocarcinoma transcript 1 (MALAT1) represent 2 distinct but interconnected areas of study in ocular research.^[9]^ Retinopathy encompasses a group of conditions that affect the light-sensitive tissue at the back of the eye, known as the retina, which include diabetic retinopathy (DR), retinoblastoma (RB), proliferative vitreoretinopathy (PVR), retinopathy of prematurity (ROP) and retinal neurodegeneration.^[10]^ Recent scientific investigations have started exploring the potential relationship between retinopathy and MALAT1, leading to considerable attention.^[9]^ Retinopathy is characterized by impaired functioning of the retina’s blood vessels, resulting in vision impairment and, in severe cases, blindness.

**Figure 1.**
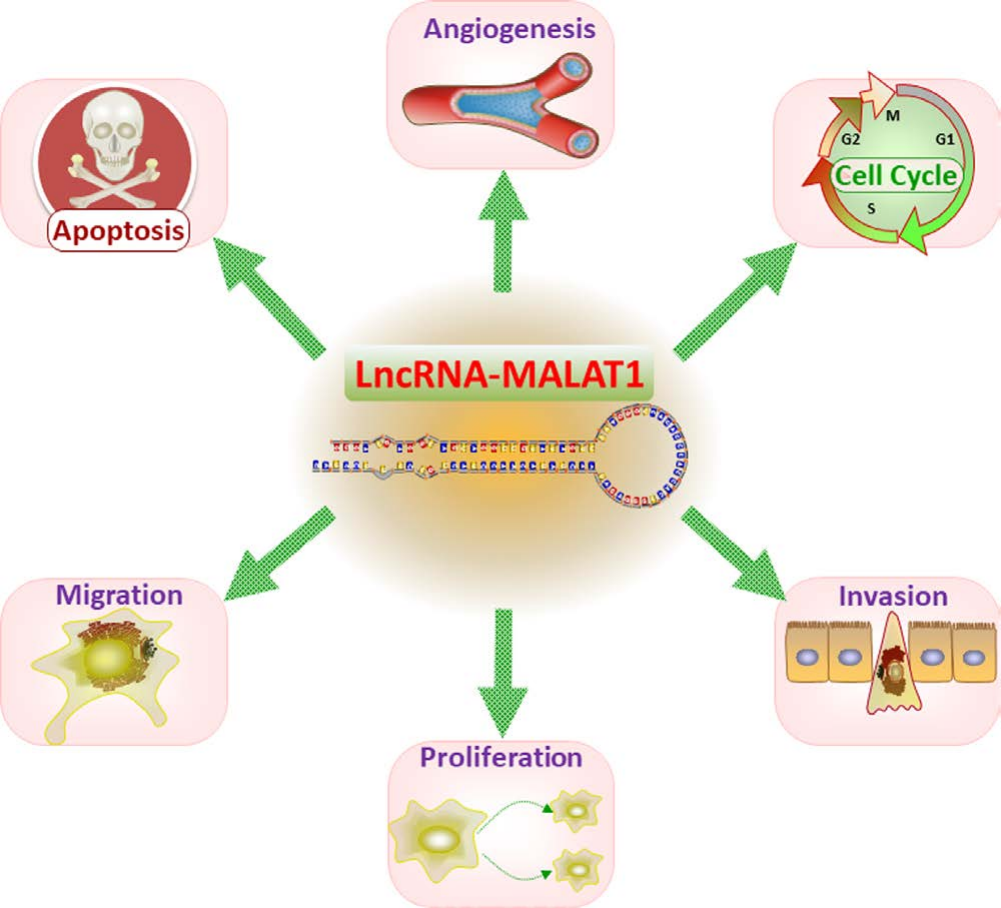
lncRNA-MALAT1 is involved in cell cycle regulation, apoptosis, angiogenesis, migration, invasion and proliferation. lncRNA = long noncoding RNA, MALAT1 = metastasis-associated lung adenocarcinoma transcript 1.

MALAT1 is 1 of the extensively studied lncRNAs known to participate in various biological processes.^[11]^ It is evolutionarily conserved and abundantly expressed in different tissues, including the eye. Studies have shown that MALAT1 plays a role in alternative splicing, contributing to gene expression regulation. Additionally, it serves as a transcriptional control mechanism and functions as a competing endogenous RNA (ceRNA) that interacts with microRNAs and modulates their activity.^[12–14]^ Dysregulation of MALAT1 has been associated with several diseases, including cancer,^[12]^ cardiovascular disorders,^[15]^ and more recently, ocular pathologies like retinopathy.^[9]^ Although retinopathy and MALAT1 initially appear to be distinct areas of research, emerging evidence suggests a potential connection between them. Understanding the roles of MALAT1 in retinopathy can shed light on the molecular mechanisms underlying the development and progression of these conditions, offering new possibilities for MALAT1 as a diagnostic and prognostic biomarker. Targeting MALAT1 could also present a novel therapeutic approach for managing retinopathy (Fig. 1).

In this review, we aim to explore the characteristics and potential links between retinopathy and MALAT1. We provide a summary of current knowledge on retinopathy, highlighting its different forms and clinical implications. Additionally, we discuss the involvement of MALAT1 in retinopathy, examining its roles in pathogenesis and its potential as a therapeutic target. By elucidating the association between retinopathy and MALAT1, this review hopes to contribute to ongoing research efforts in both fields and stimulate further investigations into their interplay.

## 2. lncRNA MALAT1 in diabetic retinopathy

DR is a significant complication of diabetes mellitus that leads to visual impairment and blindness in adults.^[16]^ The pathogenesis of DR involves increased oxidative stress in the retina, affecting the transcriptional activity of Nrf2, a critical regulator of antioxidant defense mechanisms.^[17]^ MALAT1 has been implicated in promoting neovascularization in DR, exacerbating the condition.^[16]^ MALAT1 upregulation has been associated with the progression of DR by influencing endothelial cell dysfunction and angiogenesis in the retina.^[18,19]^ Additionally, MALAT1 has been identified as an epigenetic regulator of inflammation in DR, further contributing to the pathogenesis of the disease.^[20]^ The role of MALAT1 in DR highlights its potential as a therapeutic target for managing the condition and preventing its complications.^[21]^

Traditional treatments include laser therapy and Vitrectomy surgery. New therapies like steroids or anti-Vascular endothelial growth factor (VEGF) agents intraocular injection is currently the first choice for clinical treatment, less destructive to the retina than traditional therapies, and may be useful in patients of whom unrespond to conventional therapy.^[22]^ Although New therapies have been significant advances, but up to 1/2 of patients fail to respond.^[23]^ Due to the short duration of the drug in the vitreous and neovascular eye diseases, repeated intravitreal injections of anti-VEGF drugs are often required. The Role of MALAT1 in regulation of DR has attracted great attention from researchers. The ARPE-19 cell line was used to confirm that hyperglycemia induced the expression of MALAT1 in vitro, and the corresponding expression of TNF-α, MCP-1, ICAM-1, and VEGF was also significantly increased, while the expression of these factors was significantly inhibited after silencing MALAT1. These results indicate that MALAT1 is a key regulator of retinal pigment epithelium (RPE) cell damage induced by hyperglycemia.^[24]^

Scientific researchers use microarray analysis the mouse model of streptozotocin (STZ)-induced diabetes and performed lncRNA expression profiling of retinas, turn out that around 303 lncRNAs expressed aberrantly in the retinas of early DR, of which including 214 down-regulated and 89 up-regulated, and MALAT1 was significantly up-regulated in cell model of hyperglycemia, in the aqueous humor samples, and in retinal fibrovascular membranes.^[25]^ Further research found that MALAT1 knockdown ameliorates retinal function of pericyte loss, capillary degeneration, microvascular leakage, and inflammation in diabetic rats, prevents hyper-proliferation of retinal endothelial cells (RECs) through p38 MAPK signaling.^[19,26]^ Analogously, MALAT1 expression increased in human umbilical vein endothelial cells with high glucose after 12h of incubation, associated with parallel increase in serum amyloid antigen 3, and was further accompanied by increase in mRNAs and proteins of inflammatory mediators, TNF-α and IL-6, and such cellular alterations can be prevented by MALAT1 small interfering RNA (siRNA) transfection,^[27]^ another findings indicated that MALAT1 can impact the expression of inflammatory transcripts through association with components of the polycomb repressive complex 2 in diabetics.^[20,26]^

Gene coexpression analysis revealed significant upregulation of visual perception-related genes (Pde6g, Guca1a, Rho, Sag, and Prph2) in different rodent models of Dr Utilizing bioinformatics tools based on the ceRNA hypothesis, a link between the long noncoding RNA MALAT1 and visual perception-related mRNAs was established. Specifically, MALAT1 was predicted to regulate Sag and Guca1a through miR-124-3p and Pde6g through miR-125b-5p or miR-378a-3p.^[28,29]^ Further studies demonstrated that MALAT1, acting as a ceRNA, directly binds to miR-124 and regulates the expression of Amadori-glycated albumin-induced monocyte chemotactic protein-1 (MCP-1) in retinal microglia of rats.^[30]^ Experimental evidence also suggested that MALAT1, as a ceRNA, directly binds to miR-126-5p to induce epithelial-mesenchymal transition (EMT) in RECs during proliferative DR (PDR).^[31]^

lncRNA-MALAT1 has been shown to play a significant role in regulating the proliferation, migration, and tubulogenesis of human retinal microvascular endothelial cells (HRMECs) in in vitro experiments. Knockdown of MALAT1 or upregulation of miR-203a-3p suppressed high glucose-induced proliferation, migration, and tube formation of HRMECs. Conversely, downregulation of miR-203a-3p abolished the suppressive effect of MALAT1 knockdown on HRMEC cell migration and tube formation.^[32]^ Another study demonstrated that MALAT1, as a ceRNA, could bind with miR-125b and target vascular endothelial-cadherin (VE-cadherin), Suppression of MALAT1 resulted in reduced expression of the VE-cadherin/β-catenin complex and neovascularization-related proteins,^[16]^ It was also observed that the knockdown of MALAT1 suppressed retinal endoplasmic reticulum stress.^[33]^ Additionally, the transcriptional coactivator Yes-associated protein (YAP), a major effector of the Hippo tumor suppressor pathway, was found to be highly expressed in DR mice retinas.^[34]^ YAP promoted cellular processes in retinal microvascular endothelial cells (RMECs) by upregulating MALAT1 expression and sponging miR-200b-3p. These findings suggest that YAP may play a role in DR development through the regulation of the MALAT1/miR-200b-3p/VEGF-A axis. Similarly, MALAT1 can sponge miR-205-5p to regulate VEGF-A expression.^[35,36]^

Retinal Keap1 expression levels increase in diabetes, which can mediate the nuclear movement or transcriptional activity of Nrf2, resulting in increased oxidative stress. Downregulation of long noncoding RNA MALAT1 by its siRNA prevents glucose-induced increase of Keap1, showing potential for protecting the retina from oxidative damage and preventing or slowing down Dr^[17]^ Additionally, MALAT1 downregulation protects mitochondrial homeostasis and improves capillaries.^[37]^ Knockdown of miR-320a in mouse RMECs promotes VEGF-A production, cell invasion, tube formation, and vascular permeability, while miR-320a overexpression has the opposite effect. In HG-induced mouse RMECs, MALAT1 is highly expressed while miR-320a is lowly expressed. MALAT1 acts as a ceRNA to regulate the miR-320a/hypoxia-inducible factor-1 alpha (HIF-1α) pathway, promoting the formation of retinal neovascularization in Dr^[18]^ Inflammatory response in DR includes the involvement of insulin-like growth factor-2 messenger RNA binding protein 3 (IGF2BP3). MALAT1 directly binds to IGF2BP3, increasing its expression and activating the NF-κB signaling pathway, thus promoting RPE cell damage.^[38]^

lncRNA-MALAT1 has emerged as a promising biomarker for Dr A study involving 80 patients diagnosed with type 2 diabetes and 81 healthy individuals measured the expression levels of serum miR-20b, miR-17-3p, HOTAIR, and MALAT1. The results demonstrated that decreased serum levels of miR-20b and miR-17-3p, as well as increased serum levels of HOTAIR and MALAT1, were positively correlated with the occurrence and severity of DR in diabetic patients. These findings suggest that these molecules can serve as noninvasive biomarkers for screening and early diagnosis of PDR in patients with Dr^[39]^

## 3. lncRNA MALAT1 in retinoblastoma

RB, an aggressive eye cancer that primarily affects infants and children, is 1 of the most common malignancies in this age group, drawing attention to the genetic basis of cancer. However, RB often exhibits insensitivity to chemotherapy and radiotherapy.^[40]^ Studies have shown that MALAT1 upregulation can facilitate RB cell proliferation and inhibit cell apoptosis, contributing to the progression of the disease.^[41]^ By regulating MALAT1, researchers have been able to control the growth and apoptosis of RB cells, suggesting that MALAT1 could be a potential therapeutic target for RB treatment.^[42]^ Additionally, MALAT1 has been found to aggravate human RB by affecting the expression of CCNE1 and RB protein proteins.^[43]^ Furthermore, MALAT1 has been shown to control cell cycle progression by interacting with the RB protein, indicating its role in regulating cell proliferation.^[44]^ In a study examining 4 RB cell lines, researchers observed a significant upregulation of MALAT1 expression. Subsequent experiments revealed that knockdown of MALAT1 led to decreased levels of autophagy-related proteins LC3-II and Beclin1, while increasing levels of p62 protein. Conversely, under starvation stimulation, the opposite effect was observed. Additionally, the study confirmed the reciprocal regulation between MALAT1 and miR-124, and demonstrated that Syntaxin17 (a soluble NSF attachment protein receptor of the autophagosome) directly binds to miR-124 to regulate autophagy in retinoblastoma cells. Conversely, miR-124 inhibits STX17 expression. In summary, MALAT1 inhibition weakens autophagy in RB cells through miR-124-mediated regulation of STX17.^[45]^

Coincidentally, several studies have implicated the involvement of the MALAT1-miR-124-ERK/MAPK and Wnt/β-catenin signaling pathways in RB, Silencing of MALAT1 has been found to promote apoptosis and inhibit cell migration, viability, and invasion in RB.^[46]^ Additionally, other studies have demonstrated similar conclusions involving different microRNAs such as miR-20b-5p targeting signal transducer and activator of transcription 3,^[43]^ as well as MALAT1 acting as a ceRNA to inhibit miRNA-655-3p expression, ultimately upregulating ATPase Family AAA Domain Containing 2 (ATAD2), a downstream target of miR-655-3p, and playing an oncogenic role in RB.^[47]^ Furthermore, bioinformatic analyses have proposed that MALAT1 may regulate BUB1, a mitotic checkpoint serine/threonine kinase, through miR-495-3p, suggesting a potential role in the development of RB.^[48]^ In a previous study, MALAT1 was found to act as a ceRNA to bind to miRNA-598-3p, leading to PI3K/AKT pathway activation, promoting cell proliferation, and inhibiting apoptosis in RB.^[41]^

In contrast, a 2-year study conducted on 98 RB patients revealed low serum MALAT1 expression in RB. In vitro data analysis further demonstrated that overexpression of MALAT1 promoted cell apoptosis, inhibited cell growth, down-regulated Bcl-2 protein expression, and up-regulated the expression levels of cleaved caspase-3, cleaved caspase-9, and Bax.^[42]^

## 4. lncRNA MALAT1 in retinopathy of prematurity

Improved medical technology and increased access to medical resources have significantly improved the survival rate of very low birth weight infants. However, as a consequence, there has been a notable rise in the incidence of ROP.^[49]^ While there have been advancements in understanding the pathophysiology of ROP and its management, challenges still persist, particularly in regions with limited medical resources. Such regions often face a shortage of specialized ophthalmologists and lack adequate examination equipment, resulting in difficulties in early diagnosis and treatment, consequently, researchers are actively seeking simple, accurate, and effective diagnostic and treatment approaches for ROP.^[50]^

Retinal neovascularization (RNV) is the characteristic pathological manifestation of ROP. Due to its important role in mediating angiogenesis, the involvement of lncRNA MALAT1 in ROP has been extensively investigated. In a study using an oxygen-induced retinopathy (OIR) mouse model, high expression of MALAT1 mRNA was observed in the retina of OIR mice. Intravitreal injection of MALAT1 siRNA significantly reduced the degree of retinopathy compared to the OIR control group, leading to decreased protein and mRNA expression levels of CCN1, AKT, and VEGF. These findings highlight the potential role of MALAT1 in RNV formation during ROP.^[51]^

Microarray analysis was employed to examine the expression of lncRNAs, miRNAs, and mRNAs in mice with ROP. The findings revealed that MALAT1 acts as a direct sponge for mir-124-3p. Downregulation of MALAT1 resulted in reduced expression of Early Growth Response Protein-1 (EGR1). Moreover, in a mouse model of OIR, inhibition of MALAT1 led to a significant suppression of RNV formation.^[52]^

## 5. lncRNA MALAT1 in proliferative vitreoretinopathy

PVR is a clinical syndrome mainly attributed to retinal tear, retinal hole, or complications following retinal surgical repair. It is characterized by various forms of retinal traction and subsequent formation of proliferative membranes caused by cells or inflammatory factors. Effective relief of retinal traction and prevention of membrane formation are crucial in PVR surgery to prevent recurrence.^[53]^

Microarray analysis of epiretinal membranes (ERMs) in patients with PVR revealed abnormal expression of 78 lncRNAs, with 48 up-regulated and 30 down-regulated. Specifically, MALAT1 expression was significantly up-regulated in the peripheral blood of PVR patients, which then decreased significantly after surgery. These findings suggest that MALAT1 may play a role in the pathological process of PVR and ERM formation, potentially serving as a prognostic indicator.^[54]^

EMT plays a crucial role in the pathogenesis of PVR.^[55]^ In an in vitro experiment using ARPE-19 cells, it was confirmed that inhibition of MALAT1 could attenuate the effects on TGF-β signaling and its downstream pathways, ultimately reducing EMT in RPE cells.^[56,57]^ Additionally, MALAT1 has been observed to affect the cell cycle by upregulating platelet-derived growth factor-BB (PDGF-BB), leading to a transition of vascular smooth muscle cells from a differentiated to a proliferative phenotype, contributing to various vascular diseases. Furthermore, MALAT1 acts as a ceRNA by sponging miR-142-3p, consequently regulating the expression of the autophagy-related 7 (ATG7) gene.^[58]^

## 6. lncRNA MALAT1 in retinal neurodegeneration

The nervous and vascular systems share common regulatory mechanisms that are crucial for maintaining normal functional activity. Conditions such as ischemia, inflammation, and elevated intraocular pressure can result in irreversible damage to retinal ganglion cells (RGCs), ultimately leading to complete blindness. Given the established role of MALAT1 in microvascular dysfunction, researchers sought to investigate its potential involvement in retinal neurodegeneration. In experiments using animal models with optic nerve transection, upregulation of MALAT1 expression was observed in the retina, Müller cells, and RGCs. Furthermore, it was discovered that MALAT1 regulates the function of Müller cells and RGCs through the action of cyclic AMP response element binding protein (CREB). Inhibition of CREB signaling disrupted the abnormal cellular activities induced by MALAT1 overexpression. Additionally, inhibition of MALAT1 signaling resulted in severe retinal neurodegeneration.^[59]^ Furthermore, single-cell RNA sequencing analysis conducted on retinal tissue from 3 human donors demonstrated a time-dependent disappearance of MALAT1 expression specifically in rod cells, with higher expression levels observed in rod cells compared to other retinal cell types. This finding suggests a potential significant association between MALAT1 and photoreceptors, underscoring its relevance to the human visual system.^[60]^ Additionally, in a study involving diabetic neurodegeneration in mice, the inhibition of MALAT1 was found to mitigate retinal photoreceptor damage and subsequently delay the progression of diabetic neurodegeneration.^[61]^

In contrast, a study focusing on glaucomatous ocular hypertension demonstrated a significant decrease in MALAT1 expression and a notable increase in miR-149-5p expression in a glaucoma model subjected to high-pressure conditions in vitro. Moreover, the study revealed that overexpression of MALAT1 or inhibition of miR-149-5p counteracted the inhibitory effect of RGCs on cell proliferation and the promotion of apoptosis following high-pressure culture. In summary, these findings suggest that MALAT1 may offer protective benefits for RGCs in glaucoma in vitro (Table 1).^[62]^

**Table 1:** lncRNA MALAT1 plays crucial mechanistic roles in the pathogenesis of various ophthalmic diseases.

Mechanistic role	Ophthalmic disease	Key findings	References
Regulation of gene expression	Diabetic retinopathy	MALAT1 upregulation promotes neovascularization and exacerbates the condition by influencing endothelial cell dysfunction and angiogenesis.	^[16,18,25]^
Promoter of inflammatory response	Diabetic retinopathy	MALAT1 identified as an epigenetic regulator of inflammation, further contributing to DR’s pathogenesis.	^[20]^
Modulation of cell apoptosis	Age-related macular degeneration	MALAT1 implicated in regulating RPE cell damage induced by hyperglycemia through the p53 signaling pathway.	^[24]^
Interaction with MicroRNA (miRNA)	Glaucoma	Acts as a ceRNA, sequestering miRNAs targeting genes involved in RGC survival and axonal health.	^[27,30,31]^
Epigenetic regulation	Dry eye syndrome	Contributes to the epigenetic regulation of genes associated with tear production and ocular surface maintenance.	^[59]^
Impact on proliferation and migration	Human retinal microvascular ECs	Knockdown or overexpression suppresses/inhibits high glucose-induced proliferation, migration, tube formation in HRMECs.	^[32]^
Regulation of neovascularization	Proliferative vitreoretinopathy	Significantly up-regulated in peripheral blood of PVR patients; decreased post-surgery suggests role in pathology.	^[54]^
Controlling cell cycle and EMT	Proliferative vitreoretinopathy	Attenuates the effects on TGF-β signaling pathways, reducing EMT in RPE cells.	^[55]^
Protection against neurodegeneration	Retina (ischemia, inflammation)	Upregulates expression in retina, Müller cells, and RGCs. Inhibition mitigates photoreceptor damage.	^[58,59]^
Influence on autophagy	Retinoblastoma	Inhibition weakens autophagy in RB cells through miR-124-mediated regulation of STX17.	^[44,58]^
Regulation of visual perception-related genes	Diabetic retinopathy	Significant upregulation of visual perception-related genes (Pde6g, Guca1a, Rho, Sag, Prph2). Link between MALAT1 and mRNAs established via ceRNA hypothesis.	^[28]^
Modulation of oxidative stress	Diabetic retinopathy	Downregulation prevents glucose-induced increase of Keap1, protecting retina from oxidative damage and preventing or slowing down Dr	^[17,57]^

ceRNA = competing endogenous RNA, DR = diabetic retinopathy, EMT = epithelial-mesenchymal transition, HRMECs = human retinal microvascular endothelial cells, lncRNA = long noncoding RNA, MALAT1 = metastasis-associated lung adenocarcinoma transcript 1, PVR = proliferative vitreoretinopathy, RGCs = retinal ganglion cells.

## 7. Concluding remarks and future prospects

**Figure 2.**
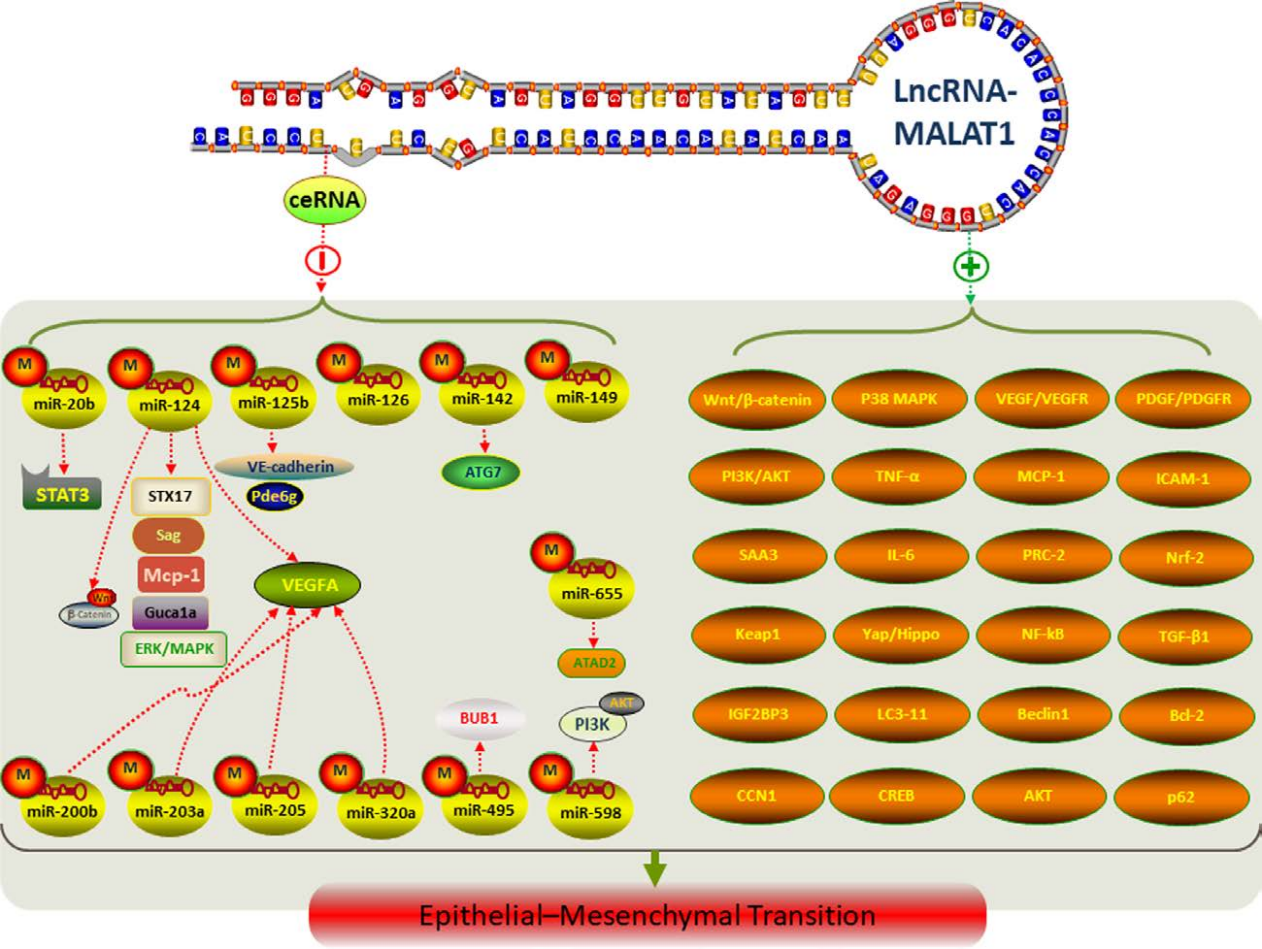
Schematic diagram of lncRNA-MALAT1 acting as ceRNAs and its involvement in the EMT process of retinopathy and regulating the corresponding signaling pathway factors. ceRNA = competing endogenous RNA, lncRNA = long noncoding RNA, MALAT1 = metastasis-associated lung adenocarcinoma transcript 1, EMT = epithelial-mesenchymal transition.

Understanding the functions and mechanisms of long noncoding RNAs (lncRNAs) is of utmost importance for the development of effective diagnostic and therapeutic strategies in the field of ocular disorders. Gene therapy has shown promising results in treating hereditary ocular disorders, including retinitis pigmentosa and glaucoma, offering targeted treatments with long-lasting effects. SiRNA-based drugs have also emerged as a potential therapeutic approach for ocular disorders. Ongoing research suggests that lncRNAs could serve as valuable tools for diagnosis and prediction of ocular disorders, potentially surpassing conventional markers in their predictive value. While the study of lncRNAs in ocular disorders is still in its early stages compared to other areas, their essential roles in various ocular diseases highlight their potential as therapeutic targets and predictive biomarkers. However, several aspects of lncRNA functions and mechanisms still require further exploration, posing challenges to their clinical implementation. Continued investigations are needed to fully unlock the potential of lncRNAs in ocular disorders and to facilitate their translation into clinical practice (Fig. 2).

The ultimate goal for ophthalmology researchers is to explore and implement the clinical application of lncRNAs in the treatment of related diseases continues. SYL040012, a glaucomatous siRNA, has been developed to specifically inhibit β2 adrenergic receptors, reducing aqueous humor production in the ciliary body and consequently lowering intraocular pressure. Preclinical animal experiments have demonstrated that SYL040012 exhibits favorable systemic and local tolerance with long-term control of intraocular pressure.^[63]^ The BamosiranTM, known by the trade name SYL040012, is a novel ophthalmic medication that offers advantages over traditional beta-blockers by exhibiting reduced systemic side effects. Currently undergoing phase III clinical trials, it holds promising potential and is anticipated to receive FDA approval in the near future.^[64]^ Despite the escalating incidence of dry eye disease (DED), attempts have been made to address ocular discomfort and pain through the development of a siRNA known as Tivanisiran. However, the pivotal Phase 3 trial assessing the efficacy of Tivanisiran did not meet the predetermined endpoints.^[65]^ However, participants treated with tivanisiran demonstrated significant improvements in pain and a reduction in central corneal damage, indicating clinically relevant benefits. This treatment may still offer significant benefits for individuals with mild or moderate DED.^[66]^ Sepofarsen, an antisense oligonucleotide, is currently being investigated for the treatment of Leber congenital amaurosis type 10 (LCA10), a retinal disease that causes childhood blindness.^[67]^ A 1-year multicenter clinical observation has revealed significant improvement in visual acuity and retinal sensitivity (post hoc analysis), supporting its potential efficacy. As a result, sepofarsen is currently undergoing further clinical development for LCA10.^[68]^ The development and utilization of these drugs highlight the significant potential and value of lncRNAs in the treatment of various diseases, which should not be overlooked.

Since its discovery, the MALAT1 has been extensively studied in various types of cancer, such as breast, cervical, colorectal, gallbladder, lung, ovarian, pancreatic, prostate, and hepatocellular carcinoma. Its ability to modulate cancer-related pathways, aid in disease diagnosis, predict prognosis, and serve as a potential therapeutic target has been well-documented.^[69]^

Through continued investigation, additional unanticipated roles of MALAT1 are likely to be uncovered. A notable study investigating the pathogenesis of diabetic wounds uncovered that MALAT1 activates TGF-β1-mediated EMT by promoting the upregulation of ZEB1 expression.^[57]^ In an animal experiment examining the effects of aging, the 3’ isoform of MALAT1 exhibited an age-related increase in retinal oxidative stress and corneal opacity, suggesting its potential significance in investigating the process of aging. Conversely, the 5’ isoform of MALAT1 did not show the same age-related changes, indicating its divergent role in this context.^[70]^ MALAT1 can regulate the downregulation of ankyrin repeat KH domain 1 (ANKHD1) expression through the YAP1/AKT axis, resulting in increased sensitivity of colorectal cancer cells to radiotherapy.^[71]^ Studies investigating sepsis and endometriosis have consistently demonstrated a positive correlation between MALAT1 expression and the levels of proinflammatory cytokines.^[72,73]^ Interestingly, recent studies have revealed that MALAT1 functions as an inhibitory lncRNA rather than a promoter of metastasis in breast cancer. Its expression level has been found to be negatively correlated with breast cancer progression and metastatic ability, which contradicts the conclusions drawn from previous experiments that focused on the inhibition or knockdown of MALAT1.^[74]^

NcRNAs have demonstrated significant potential in diagnosing and treating a range of diseases, including cancers and inflammatory conditions. They offer distinct advantages in cancer diagnostics and prognostic assessments across various medical fields,^[75]^ while their application in ophthalmic conditions remains under investigation. ncRNAs are gaining attention as possible diagnostic markers and therapeutic agents in ophthalmology, presenting exciting opportunities for future research and development. Studies on platelet-derived extracellular vesicles (PEVs) highlight their involvement in tumor development and their capability as both diagnostic markers and therapeutic agents for cancers.^[76]^ Furthermore, exosomal ncRNAs have emerged as promising treatments for retinal vascular diseases, revealing the therapeutic possibilities of incorporating ncRNAs or their inhibitors into exosomes for combating diseases.^[77]^ The role of telemedicine in diagnosing and managing ocular disorders has been underscored, with the integration of ncRNAs into telemedicine potentially improving diagnostic precision and expanding treatment avenues for eye conditions.^[78]^ Similarly, the deployment of CRISPR/Cas9 technology in targeting cancer genes offers a simplified approach to gene therapy, which could be tailored to address ophthalmic conditions.^[79]^ Looking ahead, utilizing ncRNAs in ophthalmology could lead to enhanced diagnostic methods and the creation of innovative treatments for eye diseases.^[80]^ While research on ncRNAs in ophthalmology is in its nascent stages, it promises to revolutionize future diagnostics and treatment paradigms. Continued exploration of ncRNAs’ role in eye conditions will be crucial in overcoming current limitations and obstacles, setting the stage for groundbreaking diagnostic and therapeutic approaches in the discipline.

## 8. Literature search method

For this review, a comprehensive methodology was employed to conduct a thorough literature search across various databases, including PubMed, Google Scholar, ScienceDirect, and Web of Science. The search was not limited by study design or date, ensuring a broad inclusion of both qualitative and quantitative studies. To maximize the search effectiveness, a range of specific keywords and their variations were utilized, progressing from broad terms to more specific ones. There were no restrictions placed on the initial search.

## Acknowledgments

Approval of the Institutional Review Board. The study was approved by Hainan Medical University and the First Affiliated Hospital of Hainan Medical University.

## Author contributions

**Funding acquisition:** Xionggao Huang.

**Writing – original draft:** Gukun Yang.

**Writing – review & editing:** Gukun Yang, Xionggao Huang.
